# Identification of a novel *MET* mutation in high-grade glioma resulting in an auto-active intracellular protein

**DOI:** 10.1007/s00401-015-1420-5

**Published:** 2015-04-11

**Authors:** Anna C. Navis, Sanne A. M. van Lith, Sander M. J. van Duijnhoven, Maaike de Pooter, Bahar Yetkin-Arik, Pieter Wesseling, Wiljan J. A. J. Hendriks, Hanka Venselaar, Marco Timmer, Patricia van Cleef, Paul van Bergen en Henegouwen, Myron G. Best, Thomas D. Wurdinger, Bastiaan B. J. Tops, William P. J. Leenders

**Affiliations:** Department of Pathology, Radboud University Medical Center, PO Box 9101, 6500 HB Nijmegen, The Netherlands; Department of Pathology, VU University Medical Center, Amsterdam, The Netherlands; Department of Cell Biology, Radboud Institute for Molecular Life Sciences, Radboud University, Nijmegen, The Netherlands; Centre for Molecular and Biomolecular Informatics, Radboud Institute for Molecular Life Sciences, Nijmegen, The Netherlands; Department of Neurosurgery, University Hospital Cologne, Cologne, Germany; Department of Cell Biology, University of Utrecht, Utrecht, The Netherlands; Department of Neurosurgery, VU University Medical Center, Cancer Center Amsterdam, Amsterdam, The Netherlands; ThromboDx BV, Amsterdam, The Netherlands

**Keywords:** MET, Glioma, Mutation, Protein localization, Genetic deletion, Auto-active, Intracellular location, Biomarker

## Abstract

MET has gained interest as a therapeutic target for a number of malignancies because of its involvement in tumorigenesis, invasion and metastasis. At present, a number of inhibitors, both antibodies against MET or its ligand hepatocyte growth factor, and small molecule MET tyrosine kinase inhibitors are in clinical trials. We here describe a novel variant of MET that is expressed in 6 % of high-grade gliomas. Characterization of this mutation in a glioma cell line revealed that it consists of an intronic deletion, resulting in a splice event connecting an intact splice donor site in exon 6 with the next splice acceptor site being that of exon 9. The encoded protein lacks parts of the extracellular IPT domains 1 and 2, encoded by exons 7 and 8, resulting in a novel pseudo-IPT and is named MET^Δ7−8^. MET^Δ7−8^ is located predominantly in the cytosol and is constitutively active. The auto-activating nature of MET^Δ7−8^, in combination with a lack of transmembrane localization, renders MET^Δ7−8^ not targetable using antibodies, although the protein is efficiently deactivated by MET-specific tyrosine kinase inhibitors. Testing of MET-expressing tumors for the presence of this variant may be important for treatment decision making.

## Introduction

The *MET* proto-oncogene (chromosome 7q31.2) encodes the tyrosine kinase membrane receptor MET (also called Scatter Factor Receptor), which is essential during development. Signaling from the receptor controls epithelial-to-mesenchymal transition (EMT) of myogenic precursor cells during differentiation into skeletal muscle cells [5], a process that involves migration over long distances in the embryo. In adults, MET is involved in tissue regeneration upon injury [6].

MET is produced as a glycosylated single-chain precursor protein of ~190 kDa which, during transport to the membrane, undergoes furin-mediated cleavage in the *trans*-Golgi network [8, 29]. The resulting mature receptor consists of an extracellular 50 kDa α-chain, covalently attached via a disulfide bond to a membrane-spanning 140 kDa β-chain [20, 56]. The extracellular segment consists of an N-terminal Sema domain that is involved in ligand binding, a small cysteine-rich domain, and four IPT (Immunoglobulin-like fold shared by plexins and transcription factors) domains, which connect the Sema and cysteine-rich domains with the C-terminal β-subunit [18].

Upon binding of the ligand hepatocyte growth factor (HGF, scatter factor), receptor dimerization occurs followed by trans-phosphorylation in the intracellular tyrosine kinase (TK) domain at tyrosine (Y) residues 1230, 1234 and 1235 [14, 34]. The TK domain subsequently induces auto-phosphorylation of Y1349 and Y1356, which act as docking sites for signal transduction molecules including GAB1, GRB2, phospholipase-C and SRC [50]. Phosphorylated GAB1 interacts with molecules like PI3-K and SHP2, which together induce several downstream signaling pathways. MET signaling is mediated by, among others, the PI3-K/AKT and RAS/MAPK pathways, which induce cell cycle progression, survival, cytoskeletal changes and invasion (reviewed in [17]). In addition to its role downstream of HGF, MET can also be involved in signaling of other transmembrane receptors, including VEGFR2, CD44v6, EGFR and Plexin B1 [19, 26, 35, 44]. Upon ligand-induced receptor dimerization, MET is internalized via endocytosis and may be recycled [27]. Phosphorylation of Y1003 in the juxtamembrane (JM) domain of the receptor leads to ubiquitination and subsequent proteasomal degradation [25]. Thus, levels of MET in the cell are tightly regulated.

Aberrant activation of MET signaling is a tumor-promoting event in a variety of malignancies and can be induced by several mechanisms, including alternative mRNA splicing, exon skipping and crosstalk with other receptors [13]. In high-grade gliomas, the frequently occurring oncogenic EGFR mutant EGFRvIII can induce overexpression of both MET and HGF, a process that is balanced by wild-type EGFR activation [33]. *MET* amplifications have been found in a number of tumor types including glioblastoma (GBM) [9, 10] and missense mutations in the Sema, the TK and the JM domain have been reported to affect HGF binding, kinase activation and receptor degradation, respectively [1, 30, 32, 36, 38, 43, 48, 49]. Recently, gene fusions between the protein tyrosine phosphatase *PTPRZ1* and *MET,* resulting in constitutive activation of MET, were described in 16 % of secondary GBMs [2]. Activation of MET signaling has been proposed as a mechanism of resistance to EGFR inhibitors, likely a result of the similarities in downstream signaling events from both receptors [3].

The significant role that MET plays in tumor progression and metastasis has made it a prime therapeutic target in oncology. MET tyrosine kinase inhibitors and therapeutic antibodies against the extracellular domain of MET and against HGF, all preventing HGF-mediated MET activation, are currently in clinical trial (www.clinicaltrials.gov). In a previous study, we have shown that the combined VEGFR2/MET tyrosine kinase inhibitor cabozantinib (XL-184, CoMETRIQ) potently inhibits MET phosphorylation, cell proliferation and migration and consequently prolongs survival of mice carrying orthotopic E98 glioma xenografts [42]. Here, we identify a novel intragenic *MET* deletion in E98 cells, which results in a truncated protein that is constitutively active and lacks membranous expression, thereby having important implications for therapeutic strategies targeting MET. We show that this mutation occurs in 6 % of glioblastomas and, like the EGFR mutation EGFRvIII [4], is relatively specific for this tumor type.

## Materials and methods

### Immunohistochemistry

Immunohistochemistry on formalin-fixed, paraffin-embedded (FFPE) tissue sections was performed as previously described using antibodies against MET and P-MET (clone D1C2 and D26, respectively, both CST) [42]. Antibodies were visualized via sequential incubations with biotinylated secondary antibodies, avidin–biotin complexes (Vector laboratories, Burlingame, CA, USA) and 3,3′-diaminobenzidine solution (Power-DAB, ImmunoLogic, Duiven, The Netherlands).

### Cell lines

The E98 cell line and xenograft model and genetic analysis thereof have been described before [12, 42]. E98, U87, A549, HEK-293T and TOV-112D or TOV-112D-MET cells [22] were cultured in DMEM + 4.5 g/l glucose medium (PAA Laboratories, Pasching, Austria) supplemented with 10 % fetal calf serum (FCS) (PAA) and gentamycin (40 μg/ml). All cell lines were maintained at 37 °C in the presence of 5 % CO_2_. To examine HGF-induced MET activation, E98 and A549 cells were seeded in 6 wells plates. The next day, cells were serum-starved overnight, followed by a 10 min treatment with 50 ng/ml HGF (Miltenyi Biotec, Bergisch Gladbach, Germany). In some experiments, prior to HGF incubation cells were incubated with the anti-MET llama VHH G2 [22] or cabozantinib (XL-184, Exelixis, San Francisco, CA, USA) for 60 min.

### Genetic analysis of E98

Genomic DNA from E98 cells was analyzed by semi-conductor sequencing (IonPGM, Life Technologies) using the comprehensive cancer panel (Life Technologies) that targets 409 cancer-related genes. The IonPGM E98 library generation was performed according to the manufacturer’s protocol. In short, 10 ng of DNA per pool was amplified in 21 cycles by PCR using the Ion AmpliSeqTM mastermix, followed by barcode and adapter ligation. Amplified products were purified with Agencourt AMPure XP beads (Beckman Coulter Genomics, High Wycombe, UK). The library was diluted to 20 pM. Emulsion PCR was performed using the Ion OneTouchTM 200 Template kit following the protocol of the Ion OneTouchTM System. Next, Ion Sphere Particles (ISPs) were recovered and enriched for template-positive ISPs using Dynabeads MyOne Streptavidin C1 beads (Life Technologies) in the Ion OneTouchTM ES instrument (Life Technologies). ISP enrichment was quantified using the Qubit 2.0 fluorometer (Life Technologies). Sequencing primer and polymerase were added to the final enriched spheres before loading onto an Ion 318 chip according to the Ion PGMTM 200 sequencing kit protocol. The gene copy number analysis was performed as follows. The relative number of sequence reads aligned to a specific gene was determined by dividing by the total number of aligned reads of E98, and was divided over the relative number of sequence reads of the same gene in non-neoplastic blood cells. The relative ratios are plotted in a graph based on the genomic position of the gene.

To perform confirmative mapping of the intronic deletion in *MET* on the nucleotide level, genomic DNA was isolated from E98 and U87 cells using the DNeasy Blood & Tissue Kit (QIAGEN), according to the manufacturer’s protocol. 10 ng was subjected to PCR, using primer MET1997Fw (5′-CTCCTTGGAAATGAGAGCTG-3′, forward, exon 6, genome location chr7q;g.116395517-36) and reverse primer MET2414Rev (5′-GGGATCTTCACGGTAACTG-3′, located in exon 9, CHR7q; g.116398565-45). Human genome sequence annotations are in all cases based on assembly hg19.

### FISH

Formalin-fixed, paraffin-embedded sections (4 μm) on SuperFrost glass (dried >45 min at 56 °C) were deparaffinized and rehydrated in ddH_2_O. After boiling in a microwave in sodium citrate buffer (pH 6), slides were allowed to cool to RT, washed in ddH_2_O and incubated for 5 min in 10 mM HCl. Proteins were digested with pepsin (200 U/ml, Sigma) for 15 min at 37 °C. Subsequently, slides were rinsed in 10 mM HCl and PBS and postfixed for 5 min in 1 % paraformaldehyde (PFA, Merck)/PBS. Sections were washed in ddH_2_0, dried and hybridized with 10 μl probe mix (1 μl cep7 Spectrum Green (06J37007) + 1 μl LSI MET Spectrum Red (06N05-001) + 7 μl hybridization buffer in MQ, all Vysis) under a cover slip. Sections were denatured at 80 °C for 10 min, followed by hybridization o/n at 37 °C in a Hybridizer (Dako). After removing the coverslip by soaking for 5 min in 2× SSC buffer (Maxim Biotech) at 42 °C, slides were washed 3 times in 2x SSC buffer at 73 °C, once in 2xSSC (5 min) and once in ddH_2_O. After dehydration in EtOH, slides were air-dried in the dark and mounted in Vectashield/DAPI (3 parts Vectashield/DAPI + 1 part Vectashield, all vector). Slides were analyzed on a Leica Fluorescence microscope.

### RT-PCR and cloning

RNA was isolated from cell lines E98 and TOV112D-MET [22] using TRIzol Reagent (Life Technologies) and reverse transcribed with MMLV-RT (New England Biolabs) using oligo-dT primers, according to the manufacturer’s instruction. HGF was PCR amplified from cDNA using Phusion High-Fidelity DNA polymerase (Finnzymes, ThermoFisher Scientific, Waltham, USA) and primers HGF-Fw204 (5′-CTGCAGCATGTCCTCC TGCA-3′) and HGF-Rv504 (5′-GAGGTCAAATTCATGGCCAA-3′) (30 cycles, annealing at 55 °C, 20 s; extension at 72 °C, 20 s). Control PCR reactions were performed for housekeeping gene HMBS (Hydroxymethylbilane Synthase) using primers p361-Fw (5′-TGCCAGAGAAGAGTGTGGTG-3′) and p425-Rv (5′-GTTAAGCTGCCGTGCAACATC-3′).

MET open reading frames were PCR amplified from cDNA using Phusion DNA polymerase and primers flanking start and stop codons (MET173EcoR1-Fw: 5′-CGAATTCGATAAACCTCTCATAATGAAGG-3′ and MET4421NotI-Rv 5′-AGCGGCCGCCTATGATGTCTCCCAGAAGG-3′). PCR products were purified on agarose gel, digested with NotI and cloned as blunt-NotI fragment in pIRESneo-EcoRV-NotI (Clontech Laboratories, Inc, CA, USA) to yield pIRESneo-MET and pIRESneo-MET^Δ7–8^. Full sequences were obtained by Sanger sequencing via the sequencing facility of Radboud UMC.

The extracellular parts of MET were PCR amplified from E98 and TOV-112D-MET cDNA using primers MET173EcoR1-Fw and MET3028NheI-Rv (5′-CGCTAGCCTGATCTGGTTGAACTATT AC-3′), and cloned in vector pHLsec-BAPHIS, a derivative from pHLsec-HIS (Addgene, Cambridge, MA, USA). The resulting vector adds a biotin acceptor peptide and His-tag to the carboxyterminus of the MET extracellular domains, resulting in pHLsec-MET^ED^-BAPHIS and the E98-derived variant pHLsec-MET^Δ7–8ED^-BAPHIS.

### Transfection and protein purification

pIRESneo-MET or pIRESneo-MET^Δ7–8^ were transfected into HEK-293T or TOV-112D cells in 6-well culture dishes (Greiner Bio-One, Krëmsmunster, Austria) using Fugene HD transfection reagent (Promega, Fitchburg, WI, USA) according to the manufacturers’ instructions. After 48 h, cell monolayers were washed with PBS and cell extracts prepared in RIPA buffer containing protease and phosphatase inhibitors (Cell Signaling Technology, CST, Danvers, MA, USA).

In separate experiments, pHLsec-MET^Δ7–8ED^-BAPHIS or pHLsec-MET^ED^-BAPHIS were cotransfected in a 1:1 ratio with the biotin-ligase expression construct pDISPLAY-BirA-ER (Addgene) in HEK293T cells and cells were cultured in the presence of 10 μM biotin (Sigma-Aldrich, St Louis, MO, USA). After 48 h, cytosolic extracts were made in RIPA buffer, and medium (1 ml) was mixed with 100 μl Ni–NTA Sepharose slurry (IBA, Goettingen, Germany). After a 1 h incubation at 4 °C, Ni-beads were washed with buffer (500 mM NaCl, 50 mM phosphate buffer, pH 7.4) and loaded onto a poly-prep column (Bio-Rad, Hercules, CA, USA). After washing off specifically bound proteins with 2 ml 10 mM imidazole, His-tagged an biotinylated MET extracellular domains were eluted with 0.5 ml 0.5 M imidazole, and dialysed o/n at 4 °C to 50 mM TRIS/150 mM NaCl pH 7.5.

### MET downregulation via short hairpin RNAs

To create MET knockdown constructs, shMET1 (GTATGTCCATGCCTTTGAA) and shMET2 (GTATGTCCATGCCTTTGAA) oligonucleotide heteroduplexes were ligated in pENTR/U6 vector and subsequently Gateway-cloned into pLenti6/BLOCK-iT-DEST-TagRFP according to the manufacturer’s protocols (Invitrogen). Generation of lentiviruses was done in HEK293FT cells as described previously [7].

### Protein analysis

RIPA extracts (20–40 µg protein per lane) and purified His-tagged proteins from conditioned media (equivalent of 20 µl conditioned medium) were subjected to PAGE on 8–10 % polyacrylamide gels and western blotting according to standard procedures. Nitrocellulose blots (Whatman Optitran BA-S85, GE Healthcare, Little Chalfont, UK) were blocked with PBS/Odyssey Blocking Buffer (LI-COR Biosciences, Lincoln, NE, USA) (1:1), followed by overnight incubation with primary antibodies at 4 °C. Antibodies used were against MET N-terminus (clone EP1454Y, Epitomics, Abcam, Cambridge, UK), MET C-terminus (clone D1C2), phosphorylated (P)-MET (Y1234/1235, clone D26), P-AKT (S473, clone D9E), P-ERK1/2 (T202/Y204, clone 20G11) (all CST), GAPDH (clone 6C5, Abcam), and α-tubulin (clone 236-10501, Molecular Probes, Life Technologies). Biotin groups and primary antibodies were visualized using, respectively, streptavidin-680 (Molecular Probes, Life Technology) and appropriate secondary antibodies [goat-anti-rabbit-IRDye800 (Rockland Immunochemicals, Gilbertsville, PA, USA) or Alexa Fluor 680 goat-anti-mouse IgG (Molecular Probes, Life Technologies)]. Blots were scanned on the Odyssey imager (LI-COR Biosciences).

### Protein domain modeling

A homology model for the new hybrid IPT-domain using the WHAT IF & YASARA Twinset was generated [31, 60]. We used the experimentally solved 3D structure 2uzx, which contains the human tyrosine kinase MET. The first 60 residues of the hybrid domain are identical to this structure, whereas the following 38 residues were modeled based on homology between the 2 domains.

### Confocal microscopy

E98 and U87 cells, grown on Nunc Lab-Tek chamber slides (Sigma-Aldrich) to 40 % confluence, were fixed with 2 % PFA in 0.1 M phosphate buffer (pH 7.4) for 15 min at RT, followed by three washes with PBS, 30 min glycine incubation (100 mM in PBS) to quench PFA-induced autofluorescence, three PBS washes and permeabilization with digitonin for 10 min (100 μM in PBS). Aspecific binding was blocked by incubation with 20 % normal goat serum in PBS/1 %BSA for 20 min, and cells were incubated overnight at 4 °C in a humidified chamber with PBS/1 %BSA containing (combinations of): mouse monoclonal anti-CD44 antibody (as a surface marker, clone Hermes-1, ThermoFisher Scientific); rabbit anti-MET (clone D1C2, CST); rabbit anti-P-MET (clone D26, CST); mouse anti-EEA-1 (clone 14/EEA1, BD Biosciences, early endosome marker); anti-CLIMP-63 (clone G1/296, rough ER marker, a kind gift of J. Fransen). Primary antibodies were detected using Alexa Fluor 488- or 594-labeled secondary goat-anti-mouse and Alexa Fluor 488- or 568-labeled goat-anti-rabbit IgGs (all Life Technologies), 1:200 diluted in PBS/1 %BSA. Q-nuclear deep red (1:200, Life Technologies) was used to stain nuclei, and cells were mounted in Fluoromount G with DAPI (Southern Biotech, Birmingham, AL, USA). Cells were analyzed using a confocal laser scanning microscope (Leica SP2 CLSM) and Leica confocal software.

### Biotinylation assay

E98 and A549 cells were allowed to adhere and grown to 80 % confluence in a 6-well plate (Cellstar, Greiner Bio-One, Kremsmünster, Austria). Cells were washed 3 times with ice-cold PBS and incubated for 30 min at 4 °C with 0.5 mg/ml EZ-Link Sulfo-NHS-LC-Biotin (ThermoFisher Scientific). This compound does not penetrate cells and biotinylation is therefore confined to exposed membrane proteins. In parallel, Tris-free RIPA extracts, prepared from equivalent numbers of E98 or A549 cells by repeated washing over Amicon Ultra K10 centrifugal filters (Merck Millipore, Billerica, MA, USA), were treated similarly to biotinylate all cellular proteins. The reaction was quenched by washing cells or cytosolic proteins three times with 100 mM glycine/PBS and two times with PBS (using K10 columns in the case of protein lysates). Intact biotinylated cells were then subjected to lysis with RIPA buffer. Protein concentrations in all lysates were determined using the BCA protein concentration assay (ThermoFisher Scientific). MET was immunoprecipitated from 200 µg total protein in a volume of 200 μl using anti-MET (1:50, clone D1C2, CST) for 1 h at 4 °C. Immune complexes were captured by incubation for 30 min at 4 °C with 10 μl prot A agarose slurry (Roche Diagnostics, Basel, Switzerland), followed by centrifugation (14,000 rpm, 4 min) and three PBS washes. Immune complexes were solubilized by heating (5 min 95 °C) in 30 μl 2× SDS-PAGE sample buffer (0.2 % SDS, 62.5 mM Tris–HCl pH 6.8, 10 % glycerol and 0.2 μM DTT). Samples were subjected to 10 % SDS-PAGE and western blotting as described above.

### RT-PCR analysis

Glioma tissues (*n* = 80) were obtained from the Radboud UMC and from the University of Cologne. Sarcoma (*n* = 25) and castration-resistant prostate cancer (CRPC) tissues (*n* = 43) were obtained from Radboud UMC. RNA was isolated from tissues using the mirVANA RNA isolation kit (Life Technologies) according to standard procedures. For cDNA synthesis, 1 μg RNA was reverse transcribed using the Quantitect Reverse Transcription Kit (QIAGEN, Venlo, The Netherlands) using hexanucleotides. To distinguish wtMET from MET^Δ7−8^-transcripts, a PCR was performed in AmpliTaq Gold 360 mastermix (Applied Biosystems, Life Technologies) using primer MET1997Fw (5′-CTCCTTGGAAATGAGAGCTG-3′, forward, located in exon 6), and primer MET2342Rv (5′-CAGTTGAAATGGTTTGGGCTG-3′, reverse, located in exon 9). This PCR results in a 105-bp product for MET^Δ7–8^ and a 345-bp product for wtMET. Conditions were: denaturation 95 °C, followed by 35 cycles of 95 °C denaturation; annealing at 58 °C, 30 s; elongation at 72 °C, 1 min. A final elongation step of 7 min was done at 72 °C.

## Results

### E98 MET protein is auto-activated in an HGF-independent fashion

**Fig. 1:**
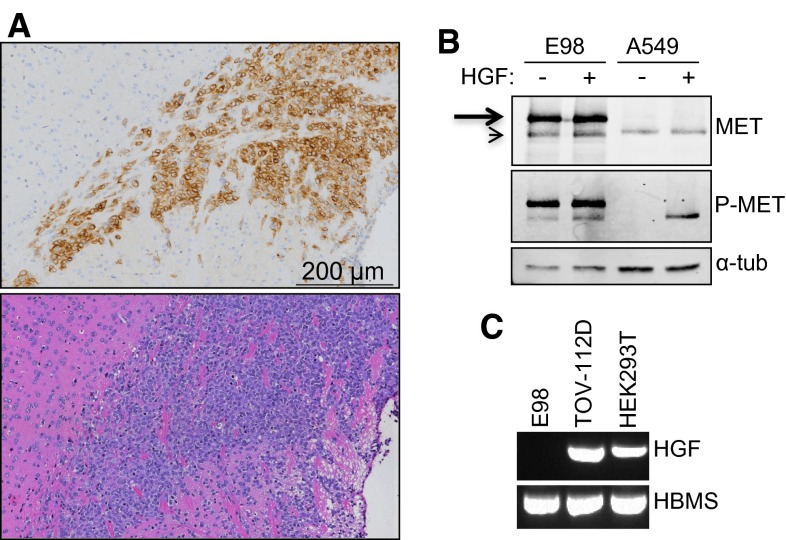
MET is constitutively activated in the E98 cell line and xenograft model. **a** Sections of an intracranial E98 xenograft were subjected to immunohistochemistry for P-MET (*upper panel*, brown staining). A matching H&E staining is shown as reference. Note that tumor shows diffuse infiltration in the brain parenchyma and that the tumor cells are highly positive for this activated MET. **b** Western blot analysis of MET expression in serum-starved E98 and A549 cells in absence of presence of HGF. Note that the processed form of MET (*arrowhead*) in E98 is somewhat smaller than that of A549 while the preform is predominantly present in E98 (*arrow*). α-Tubulin was used as a loading control. **c** RT-PCR for HGF on E98, TOV-112D and HEK-293T cell line cDNA. HMBS was used as a control housekeeping gene

Our previous experiments have shown that MET in orthotopic E98 xenografts is phosphorylated in tumor cells in a homogeneous fashion (Fig. 1a, see also [42]). In vitro, E98 cells also show high levels of phosphorylated MET when grown under serum-free conditions (Fig. 1b). HGF treatment did not further increase phosphorylation levels of MET, in contrast to A549 control cells in which HGF was required for MET activation. MET phosphorylation in E98 cells was not the result of endogenous HGF expression as revealed by RT-PCR analysis (Fig. 1c), while analysis of E98 xenograft RNA revealed the presence of mouse HGF only, as determined by Sanger sequencing (not shown). Since mouse HGF is not an activating ligand for human MET [62], we conclude that constitutive activation of MET in E98 cells and xenografts is not the result of an autocrine HGF-activation loop.

### E98 cells express a truncated version of an amplified MET gene

**Fig. 2:**
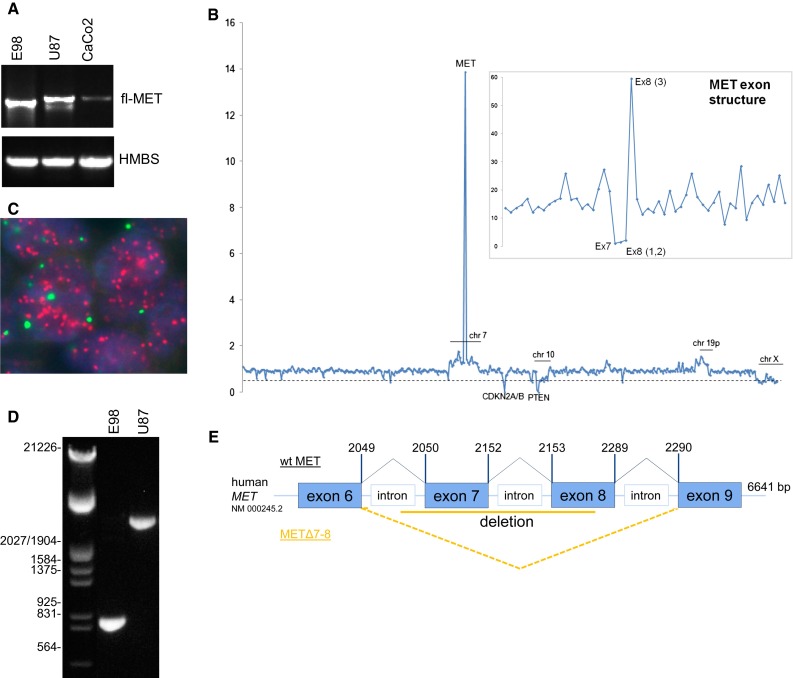
E98 MET contains an intronic deletion, resulting in a truncated transcript. **a** RT-PCR on cDNA from different cell lines using full-length MET primers, using HMBS as a housekeeping gene for reference. **b** Ion-torrent sequencing analysis exposes a high-copy amplification of MET in E98 DNA. Plotted on the *x*-axis are all 409 genes sequenced, in chromosomal order. The number of reads per gene was compared to blood-obtained DNA from a glioma patient as a reference. The *dotted line* represents the level of heterozygous losses, as can be seen for the X-chromosome (the reference blood sample was female, while the tumor DNA was male). The *inset* shows the relative number of reads per *MET* exon. Note the loss of exon 7 and part of exon 8. **c** FISH analysis of the number of *MET* (*red*) and *chr.7* centromere (*green*) copies in the genome of an E98 xenograft. **d** Amplification of *MET* using exon 6- and 9-specific primers on genomic DNA of E98 and U87 cells. MET^Δ7**–**8^ and wtMET amplification leads to 910- and 3041-bp products in E98 and U87, respectively. **e** Schematic overview of the MET^Δ7**–**8^ deletion found in E98 cDNA. Splicing of exon 6 to exon 9 is indicated with the *dotted orange lines*. The *orange solid line* represents the deletion

To examine the underlying mechanism of MET auto-activation in this model, we PCR-cloned MET cDNA from E98 cells, using primers flanking the open reading frame (ORF, NM_000245.2). The MET PCR product from E98 was 240 bp smaller than that from U87 and CaCo2 cDNA (Fig. 2a), and Sanger sequencing analysis revealed an in-frame deletion of nt 2050–2289 in the coding sequence, corresponding to exons 7 and 8. The same transcript was also found in E98 xenografts (not shown). Genomic analysis of E98 cells using semi-conductor sequencing revealed high copy *MET* amplification (Fig. 2b). Average amplification of *MET* (except for exons 7/8) was about 14-fold. FISH analysis using probes specific for MET and chromosome 7 centromere confirmed the amplification (Fig. 2c). Consistent with Sanger sequencing, amplicons in exon 7 and the first part of exon 8 were absent (see insert in Fig. 2b), suggesting that the lack of exons 7 and 8 results from a genomic rearrangement (similar to the EGFR variant III [15]), instead of alternative splicing. PCR on genomic DNA using exon 6- and 9-specific primers resulted in amplification of a 910-bp fragment (predicted size from the wild-type allele, present in U87, is 3014 bp, see Fig. 2d). Sequencing of this product revealed an intronic deletion of 2114 bp (between position g.116 395 653 located in intron 6, and 116 397 766, located in exon 8). This deletion results in an intact splice donor site from exon 6, juxtaposed to the exon 9 splice acceptor site, and explains the lack of exons 7 and 8 in the resulting mRNA (Fig. 2e). Of note, a wt*MET* allele could not be detected in E98 cells (Fig. 2d).

**Fig. 3:**
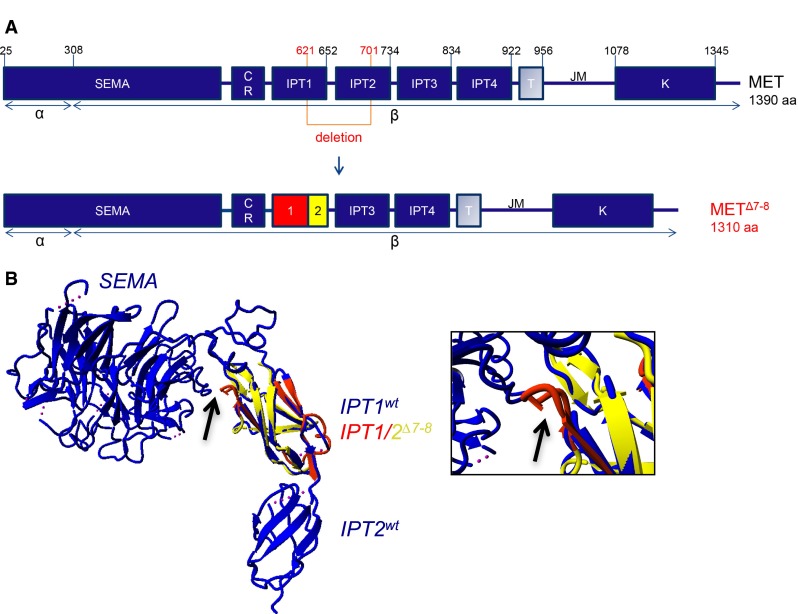
Predicted protein structure of MET^Δ7**–**8^. **a** Schematic overview of MET and MET^Δ7–8^. The *red lined box* represents the deletion, comprising parts of the IPT1 and 2 domains (represented by the *red* and *yellow boxes,* respectively, in MET^Δ7–8^). **b** Yasara modeling of the remaining IPT1 and 2 domains in MET^Δ7**–**8^ was performed based on wtMET IPT1. The wtMET SEMA domain and IPTs are shown in *blue*, with the novel putative IPT domain consisting of IPT1 (*red*) and 2 (*yellow*) of MET^Δ7**–**8,^ superimposed on wtMET. An enlargement of the fusion part in the overview figure (*arrow*) is shown in the *right panel*. The *arrow* indicates the additional loop in *red* that is formed by the novel IPT domain

In the MET protein, this rearrangement leads to loss of the C-terminus of IPT1 and the N-terminus of IPT2 (Fig. 3a). Yasara modeling, using X-ray crystallographic data of the MET ectodomain, predicts the formation of a novel IPT, composed of the remaining parts of IPT1 and 2. Interestingly, in the new IPT1/2 fusion, a small stretch of 5 extra amino acids loops out towards the Sema domain (Fig. 3b, arrow).

### MET^Δ7–8^ is aberrantly processed

**Fig. 4:**
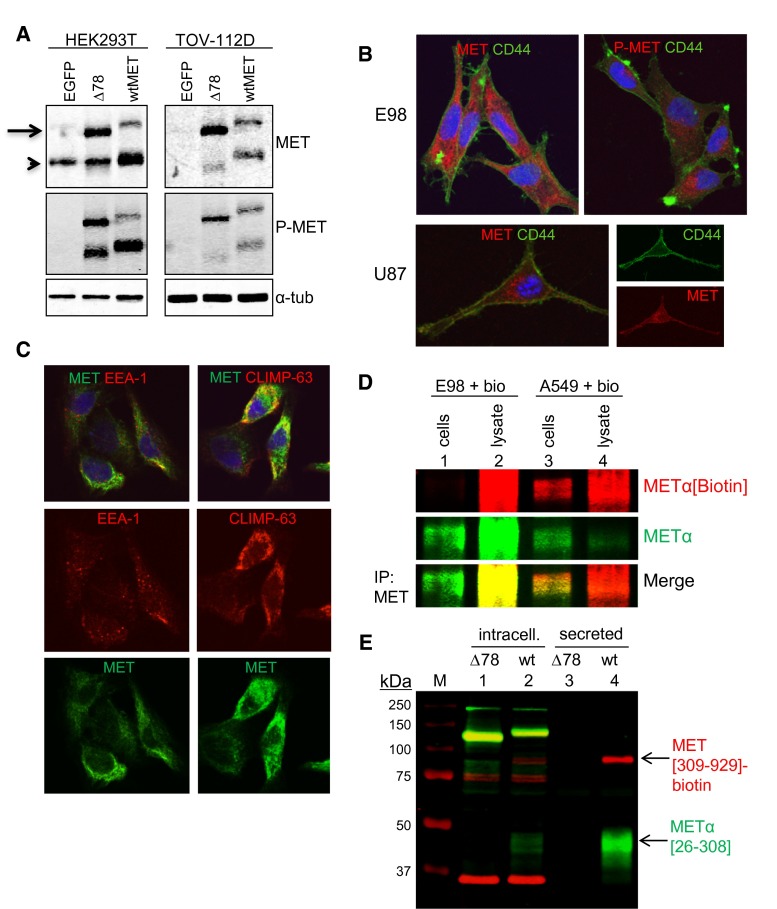
Aberrant processing and localization of MET^Δ7**–**8^. **a** Western blot analysis of MET expression in HEK-293T and TOV-112D cells, transfected with pIRESneo-MET or pIRESneo-MET^Δ7**–**8^. Note that HEK-293T cells have endogenous MET expression. As a loading control, α-tubulin was used. **b** Analysis of MET localization in E98 by confocal microscopy, using CD44 as a membrane marker (*green*) in combination with C-terminal MET antibodies (*red*), or phosphorylated MET (1234/1235, *red*) as indicated. U87 cells were also analyzed for MET/CD44 colocalization for comparison. Q-nuclear deep red stain was used to stain all nuclei. **c** Subcellular localization of MET (*green*) with early endosome and RER markers EEA-1 and CLIMP-63 (both in *red*) in E98 cells. **d** Western blots of biotinylated intact E98 and A549 cells or of biotinylated cell lysates. MET was immunoprecipitated and analyzed on western blot for biotinylation, as described in the materials and methods section. The α-chain of MET is shown. **e** His-tagged ectodomains of both wild-type MET and MET^Δ7**–**8^, biotinylated via a biotin-acceptor peptide at the carboxyterminus, were expressed in HEK-293T cells. Ni-purified culture media and extracts of the transfected cells were used to analyze MET localization. Both fractions were subjected to western blotting using N-terminal MET antibodies, recognizing both the preform and the cleaved α-portion of the ectodomain, and directly labeled streptavidin to visualize the preform and the β-chain. GAPDH was used as a loading control for the RIPA extracts (*red* signal at 36 KDa)

MET is synthesized as a 190-kDa precursor protein which is proteolytically cleaved by furin between residues 307 and 308, to yield an extracellular α-chain of approximately 45 kDa, covalently linked via a disulfide bridge to the transmembrane β-chain. Reducing SDS-PAGE, followed by Western blot analysis of E98 cell extracts showed that the majority of MET protein was in a 180-kDa phosphorylated form, corresponding to the uncleaved truncated preform (Fig. 1b, arrow). In contrast, in A549 cells the matured cleaved MET protein was the predominant form (arrowhead in Fig. 1b). To investigate whether this was a specific feature of E98 cells, we analyzed the protein structure in cells after transfection with the full-length cDNAs encoding MET^wt^ or MET^Δ7–8^. In both HEK-293T and TOV-112D cells, wtMET was properly processed to an α- and β-chain, indicating that these cells are not defective in furin-mediated processing. In contrast, MET^Δ7–8^ was predominantly present in the uncleaved preform in both cell types (Fig. 4a, arrow). Thus, improper MET cleavage is an intrinsic property of MET^Δ7–8^. Overexpression studies in HEK-293T and TOV-112D cells resulted in phosphorylated Y1234/1235 residues in both MET^wt^ and MET^Δ7–8^ proteins (Fig. 4a, P-MET). Because both cell lines produce HGF (Fig. 1c), this may be a result of HGF-dependent autocrine activation.

MET is cleaved by the endoprotease furin, which is localized predominantly in the *trans*-Golgi network, but also in vesicles and near the plasma membrane [8, 29, 52]. To test whether the inefficient cleavage of MET^Δ7–8^ in E98 cells is related to intracellular transport defects, we analyzed the subcellular localization of MET^Δ7–8^ in detail via confocal microscopy. MET^Δ7–8^ did not co-localize with the cell surface marker CD44 and was confined to the cytosol (Fig. 4b). In contrast, in U87 cells MET^wt^ did co-localize with CD44. Additional intracellular staining in U87 cells reflects de novo synthesized material that is being processed for constitutive secretion. Immunostainings with the early endosome marker EEA-1 and the rough endoplasmic reticulum (RER) marker CLIMP-63 suggested that MET^Δ7–8^ in E98 cells is predominantly retained in the RER (Fig. 4c).

To confirm the absence of cell surface expression of MET^Δ7–8^ on E98 cells, we labeled intact E98 cells or cell lysates with NHS-biotin and immunoprecipitated MET using specific antibodies, followed by SDS-PAGE/Western blot and staining for biotin and MET. Whereas METs’ N-terminal α-chain was readily biotinylated in E98 cell lysates, no detectable MET biotinylation occurred when intact cells were labeled (Fig. 4d, lane 1). In contrast, biotinylated MET was readily detected in A549 cells upon labeling of intact cells, as shown by the biotin-labeled α-chain (Fig. 4d, lane 3). Thus, these data confirm that MET^Δ7–8^ is predominantly localized intracellular and is poorly exposed on the cell surface of E98 cells.

To further confirm a defect in intracellular trafficking of MET^Δ7–8^, we analyzed secretion patterns of extracellular domains of wtMET or MET^Δ7–8^ (ending with residue D^929^, numbering according to MET variant 2 (NP_000236.2), containing a C-terminal biotin tag. Whereas the extracellular domain of wtMET was properly processed and secreted into the culture medium (as illustrated by the presence of the 309-929 biotinylated extracellular β-chain and the MET25-308 α-chain, Fig. 4e, lane 4), no secreted MET products were found in medium of cells, transfected with the MET^Δ7–8^ ectodomain (Fig. 4e, lane 3). Instead, all biotinylated MET products were located intracellularly (Fig. 4e, lane 1).

### 185-kDa MET^Δ7–8^ is not affected by antagonistic anti-MET antibodies but is inhibited by cabozantinib

**Fig. 5:**
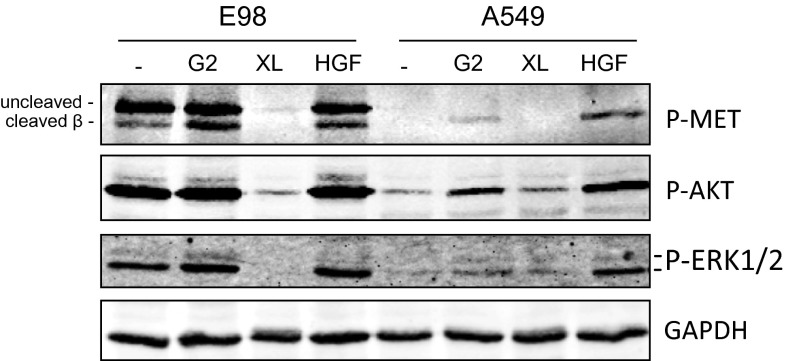
MET^Δ7–8^ is blocked by TKIs but not by inhibiting antibodies. E98 and A549 cells were left untreated or treated with the MET VHH G2, cabozantinib (XL; MET/VEGFR2 inhibitor), or HGF. Protein lysates were analyzed for MET, AKT and ERK1/2 (p42/44) phosphorylation. GAPDH was used as a loading control. Note the prominent uncleaved MET protein in E98, which is absent in A549 cells

It was previously reported that incubation of A549 cells with VHH G2, a recombinant single-domain llama antibody against MET, results in low MET activation levels, while inhibiting the strong activation which is induced by HGF [22]. To test whether and how G2 affects phosphorylation of MET^Δ7–8^, we treated serum-starved E98 cells with G2, either followed or not followed by HGF, using A549 cells as control. As shown in Fig. 5, HGF did not increase overall MET phosphorylation levels, although, interestingly, a slight activation was seen in the minority of processed β-fragment of MET. For G2, a similar effect was observed. Consistent with high overall MET phosphorylation levels in all samples, neither G2 nor HGF treatment resulted in altered levels of P-AKT and P-ERK1/2, both targets of MET. In A549 cells G2 and HGF induced MET phosphorylation, which increased levels of P-AKT and P-ERK1/2. Thus, in contrast to A549 cells that express MET^wt^, E98 cells are not responsive to antibodies against or ligands of MET. However, both E98 and A549 cells responded well to the MET tyrosine kinase inhibitor cabozantinib (Fig. 5).

### Prevalence of MET^Δ7–8^

Data mining of the COSMIC and TCGA databases did not uncover the intronic deletion in gliomas and other tumor types (not shown). Intragene deletions spanning multiple exons are, however, in general more difficult to recognize in whole-exome sequencing (WES). Furthermore, the coverage of the exact location of the deletion is poor in the mostly used WES protocols, and as can be seen in Fig. 2b, even targeted sequencing reveals the mutation only after detailed bioinformatic analysis. We, therefore, assume that detection of MET^Δ7–8^ requires dedicated PCR protocols.

**Fig. 6:**
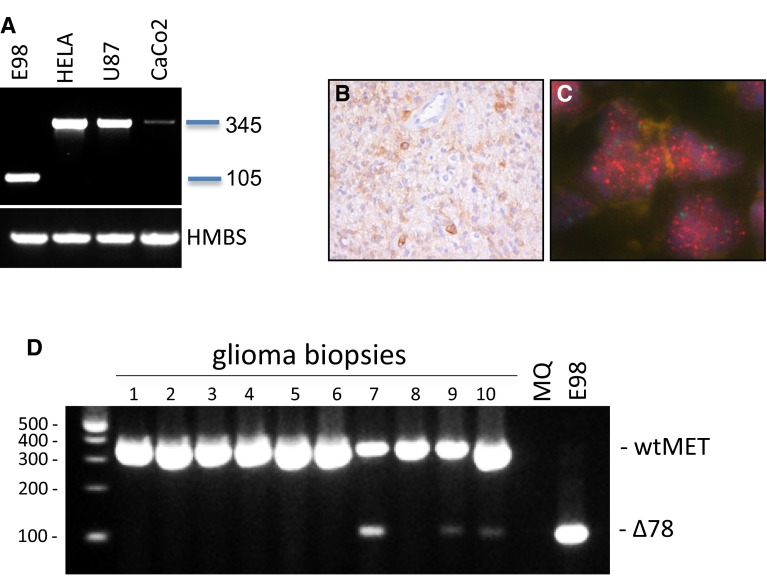
MET^Δ7–8^ is expressed in glial tumors. **a** PCR analysis of cell line cDNAs using primers located in exon 6 and exon 9. This PCR results in a product of 105 bp for the MET^Δ7–8^, while wtMET gives a 345-bp product. **b** Immunohistochemical staining for MET of the patients’ tumor from which the E98 cell line has been generated. **c** FISH analysis of the number of *MET* (*red*) and *chromosome 7* (*green*) copies in the original E98 patient tumor. **d** PCR analysis for MET^Δ7–8^ on a random set of glioma biopsies. Note that in tumors with MET^Δ7–8^ expression, also wtMET is observed

PCR with deletion-spanning primers revealed the presence of the deletion-specific 105-bp fragment in E98 cDNA, while a number of other cell lines presented with the wt 345-bp amplicon only (Fig. 6a). Since frozen material from the patient tumor that was used to generate E98 is unavailable, we could unfortunately not obtain genomic DNA and cDNA of sufficient quality to confirm the presence of the mutation in the originating tumor. We did however perform immunohistochemistry on formalin-fixed, paraffin-embedded tumor material from this patient and observed a highly heterogeneous staining for MET, with only a small percentage of strongly positive tumor cells, apparently with intracellular staining (Fig. 6b). Of note, such immunostainings cannot discriminate between MET^wt^ and MET^Δ7–8^. FISH analysis confirmed the presence of the MET amplification in the original tumor (Fig. 6c).

To investigate the prevalence of the *MET*^*Δ7*–*8*^ mutation further, we performed the exon 6–9 PCR on cDNA, generated from a series of gliomas (*n* = 102) and a number of other tumor types in which MET has been suggested to play an important role (castration-resistant prostate carcinomas, *n* = 43; Ewing sarcoma, *n* = 21; rhabdomyosarcoma, *n* = 22) [24, 28]. MET^Δ7–8^ was found in 6 out of 102 gliomas, both grade III and IV (5.8 %; 2 out of 5 anaplastic oligodendrogliomas, 2 out of 16 anaplastic astrocytomas and 2 out of 61 glioblastomas), and was not detected in the other tumor types tested (Table 1). All grade III tumors with MET^Δ7–8^ were IDH1-R132H mutated. Most tumors that contained the mutation were heterozygous, containing also the wild-type transcript (example in Fig. 6d). Further research with higher numbers of patients is needed to investigate any correlations between MET^Δ7–8^ and other molecular aberrations.

**Table 1:** MET^Δ7–8^ expression in glioma, sarcoma and CRPCs

Tumor type	Δ7/8 occurrence
Glioma	6/102 (5.8 %)
O-II	0/3
A-II	0/7
OA-II	0/5
O-III	2/5 (40 %)
A-III	2/16 (12.5 %)
OA-III	0/5
GBM	2/61 (3.3 %)
Sarcoma	0/25
Ewing tumor	0/5
Cell line	0/3
Rhabdo tumor	0/15
Cell line	0/2
CRPC	0/43

The incidence of MET^Δ7–8^ in different subtypes and malignancy grades of diffuse glioma, sarcomas and castration-resistant prostate carcinomas (CRPC)

*O-II* oligodendroglioma (WHO grade II), *O-III* oligodendroglioma (WHO grade III), *A-II* astrocytoma (WHO grade II), *A-III* anaplastic astrocytoma (WHO grade III), *GBM* glioblastoma

Sanger sequencing of the smaller PCR products of one patient with glioma confirmed the deletion of exons 7 and 8 (not shown). Of note, due to the lack of high-quality genomic DNA from these clinical samples we could not discriminate in these samples whether MET^Δ7–8^ resulted from a mutation or from exon skipping or alternative splicing, such as suggested for MET variants lacking exon 10 or 14 [37].

## Discussion

A major problem in glioma treatment is diffuse growth in the neuropil, and it has been suggested that the *MET* oncogene is causally involved in this phenotype in subsets of tumors [16]. Several signal transduction pathways that are induced by MET activation are shared with other tyrosine kinase receptors and it is increasingly recognized that activation of MET bypasses the need for EGFR activation, generating resistance to EGFR-targeted therapies [13]. Also, there is an interesting crosstalk between MET and EGFR: the EGFR variant III (EGFRvIII) stimulates expression of both MET and its ligand HGF, possibly contributing to oncogenicity of EGFRvIII [16], but this phenomenon is counteracted by EGF-mediated activation of wild-type EGFR in the same complex [33].

We previously reported that MET is crucially implicated in proliferation, survival and migration of (EGFR-negative) E98 glioma cells in vitro. To further investigate the involvement of MET in the biological behavior of E98 cells, we attempted to knock down the protein by a lentiviral shRNA approach. Attempts to generate stable MET knockdowns using 2 different MET shRNAs failed since both shRNA encoding lentiviruses were lethal to E98 cells, in contrast to control viruses (data not shown). In agreement with a crucial role of MET for cell survival, we showed that treatment with the MET tyrosine kinase inhibitor cabozantinib prolongs survival of mice carrying orthotopic E98 xenografts [42]. Based on the observation that the pattern of MET phosphorylation in E98 xenografts was remarkably homogeneous in the absence of its ligand HGF, we analyzed the MET product in E98 cells in more detail and found that the protein is expressed as a truncated product, which lacks effective furin cleavage and is predominantly retained intracellular in its active, phosphorylated form. The truncation generates a novel IPT domain consisting of a fusion between the carboxyterminus of IPT1 and the amino terminus of IPT2. Modeling of the novel fusion IPT domain suggests that it adopts a similar structure as the IPT domains in wild-type MET, although a small stretch of extra amino acid residues is accommodated in a loop, extending towards the Sema domain. Since this loop approaches the furin cleavage site in the Sema domain, it may directly affect furin cleavage. Interestingly, the colorectal adenocarcinoma cell line Lovo lacks furin protease and consequently cannot properly process MET [40]. In this cell line, the unprocessed preform is still expressed on the cell surface, binds HGF and is capable of signaling. Thus, defective processing alone does not explain intracellular retention of MET^Δ7–8^ and we therefore hypothesize that the novel IPT1-2 fusion domain in MET^Δ7–8^ is involved in defective furin cleavage, intracellular retention of the uncleaved perform and auto-activation of the uncleaved preform.

Recently, effects of various activating mutations in the MET TK domain have been analyzed in detail. These mutants are expressed on the cell surface but are subject to increased rates of turnover, resulting in accumulation in early endosomes where they can still signal [27]. MET^Δ7–8^ did not accumulate in early endosomes, suggesting that the underlying mechanism of intracellular retention is different [41]. Our confocal microscopy and biotinylation experiments strongly suggest that only the very small fraction of MET^Δ7–8^ that is cleaved reaches the cell membrane, but this fraction is insignificant with respect to the auto-active component in the cytosol.

From a genetic perspective, the *MET*^*Δ7*–*8*^ mutant resembles the auto-active EGFRvIII variant which occurs concomitant with *EGFR* amplification in 25–64 % of all GBMs [51, 59]. This variant results from a genetic deletion of exons 2–7 and its activation is also independent of the ligands EGF or TNFα. Like the *EGFRvIII* mutation [15], the MET^Δ7–8^ alteration appears to be restricted to only few cancer types, as we detected it in glioma but not in a number of other tumor types. The mutation was detected in grade III gliomas of both oligodendroglial and astrocytic origin and GBMs. Investigations of MET^Δ7–8^ expression in other tumor types is however warranted and is ongoing in our lab.

Another example of a tyrosine kinase receptor which often carries deletions in GBM is *PDGFRA*. Forty percent of the GBMs with amplified *PDGFRA* contain an in-frame deletion in the ectodomain, leading to constitutive activation of the TK domain [46]. Recently, also in pediatric high-grade gliomas, ligand-independent and tumorigenic in-frame *PDGFRA* deletion variants have been reported [47, 58]. Data on the subcellular localization of such mutated oncoreceptors are frequently lacking, and our data call for in-depth analysis of the cellular localization of receptors that are considered targetable. Interestingly, activating mutations in the RON tyrosine kinase receptor have been identified which resemble the MET^Δ7–8^ mutation in that it also involves the first IPT domain [39]. Of note, these alterations do not lead to a loss of expression at the cell surface.

Importantly, we did not detect a MET^wt^ transcript in E98 cells, in contrast to the clinical gliomas which were analyzed in this study and which all showed abundant wtMET, also in MET^Δ7–8^ tumors. We formally cannot exclude that the wild-type MET amplicons in our PCR derive from ‘contaminating’ non-neoplastic stromal cells in the tumor biopsies that were tested. There may, however, be another explanation for the relatively low levels of MET^Δ7–8^ in clinical tumors: in the patient tumor from which the E98 model was generated, a low percentage of MET-expressing tumor cells was detected, although it is impossible to determine whether these cells carry the MET^Δ7–8^ mutation because the antibodies used do not discriminate between MET and MET^Δ7–8^. It is tempting to speculate that during the generation of the E98 model, a small subset of *MET*^*Δ7*–*8*^ tumor cells in the primary tumor experienced a growth advantage, ultimately resulting in clonal outgrowth during xenograft formation. This scenario fits with the notion that clinical tumors not only show inter- but also intratumoral heterogeneity [54], and derived preclinical tumor models may only be representative for the most malignant population of tumor cells.

Expression of MET^Δ7–8^ may have important consequences for choice of therapy. MET is increasingly recognized as an important target in multiple tumor types, including glioma, and therapeutic antibodies against HGF or the HGF binding site on MET have been developed. Since HGF is not involved in MET^Δ7–8^ activation and MET^Δ7–8^ is retained intracellular, *MET*-mutated cells will not be responsive to these therapies. Indeed, we were able to show that E98 cells do not respond to the anti-MET VHH G2. Selection of *MET*-mutated cells in tumors that initially respond to antibody-based MET-directed treatment is expected to result in recurrence of treatment-resistant clones. In this respect, it will be important in future studies to assess the occurrence of the Δ7–8 mutation in paired samples of primary and recurrent tumors after MET antibody-based therapies, but also anti-EGFR therapies since cells may use MET^Δ7–8^ to bypass EGFR signaling [3, 53]. With this in mind, the use of specific tyrosine kinase inhibitors of MET may have preference over antibody-based therapy for resistant tumors, at least in the ones that are KRAS and RAF wild type, [3, 21, 57, 61] since MET^Δ7–8^ is sensitive to these inhibitors [42]. Such inhibitors have already shown to improve overall survival of patients with non-small cell lung carcinoma with MET amplification and renal papillary carcinoma with MET mutations [11, 45]. A recent clinical study with anti-MET antibody MetMab for lung cancer failed to meet the primary endpoint of prolonged survival [55]. A study of MET mutations and intracellular localization patterns in this patient group may be highly informative for future therapeutic directions [23].

In conclusion, we describe a highly active, non-ligand-dependent mutant of MET in 6 % of gliomas, which is not exposed on the cell surface and is predicted to be non-targetable with therapeutic antibodies against MET and/or HGF.

## References

[1] Asaoka Y, Tada M, Ikenoue T, Seto M, Imai M, Miyabayashi K, Yamamoto K, Yamamoto S, Kudo Y, Mohri D, Isomura Y, Ijichi H, Tateishi K, Kanai F, Ogawa S, Omata M, Koike K (2010). Gastric cancer cell line Hs746T harbors a splice site mutation of c-Met causing juxtamembrane domain deletion. Biochem Biophys Res Commun.

[2] Bao ZS, Chen HM, Yang MY, Zhang CB, Yu K, Ye WL, Hu BQ, Yan W, Zhang W, Akers J, Ramakrishnan V, Li J, Carter B, Liu YW, Hu HM, Wang Z, Li MY, Yao K, Qiu XG, Kang CS, You YP, Fan XL, Song WS, Li RQ, Su XD, Chen CC, Jiang T (2014). RNA-seq of 272 gliomas revealed a novel, recurrent PTPRZ1-MET fusion transcript in secondary glioblastomas. Genome Res.

[3] Bardelli A, Corso S, Bertotti A, Hobor S, Valtorta E, Siravegna G, Sartore-Bianchi A, Scala E, Cassingena A, Zecchin D, Apicella M, Migliardi G, Galimi F, Lauricella C, Zanon C, Perera T, Veronese S, Corti G, Amatu A, Gambacorta M, Diaz LA, Sausen M, Velculescu VE, Comoglio P, Trusolino L, Di Nicolantonio F, Giordano S, Siena S (2013). Amplification of the MET receptor drives resistance to anti-EGFR therapies in colorectal cancer. Cancer Discov.

[4] Bax DA, Gaspar N, Little SE, Marshall L, Perryman L, Regairaz M, Viana-Pereira M, Vuononvirta R, Sharp SY, Reis-Filho JS, Stavale JN, Al-Sarraj S, Reis RM, Vassal G, Pearson AD, Hargrave D, Ellison DW, Workman P, Jones C (2009). EGFRvIII deletion mutations in pediatric high-grade glioma and response to targeted therapy in pediatric glioma cell lines. Clin Cancer Res.

[5] Bladt F, Riethmacher D, Isenmann S, Aguzzi A, Birchmeier C (1995). Essential role for the c-met receptor in the migration of myogenic precursor cells into the limb bud. Nature.

[6] Borowiak M, Garratt AN, Wustefeld T, Strehle M, Trautwein C, Birchmeier C (2004). Met provides essential signals for liver regeneration. Proc Natl Acad Sci.

[7] Bourgonje AM, Navis AC, Schepens JT, Verrijp K, Hovestad L, Hilhorst R, Harroch S, Wesseling P, Leenders WP, Hendriks WJ (2014). Intracellular and extracellular domains of protein tyrosine phosphatase PTPRZ-B differentially regulate glioma cell growth and motility. Oncotarget.

[8] Bresnahan PA, Leduc R, Thomas L, Thorner J, Gibson HL, Brake AJ, Barr PJ, Thomas G (1990). Human fur gene encodes a yeast KEX2-like endoprotease that cleaves pro-beta-NGF in vivo. J Cell Biol.

[9] Cancer Genome Atlas Research N (2008). Comprehensive genomic characterization defines human glioblastoma genes and core pathways. Nature.

[10] Cancer Genome Atlas Research N (2014). Comprehensive molecular profiling of lung adenocarcinoma. Nature.

[11] Choueiri TK, Vaishampayan U, Rosenberg JE, Logan TF, Harzstark AL, Bukowski RM, Rini BI, Srinivas S, Stein MN, Adams LM, Ottesen LH, Laubscher KH, Sherman L, McDermott DF, Haas NB, Flaherty KT, Ross R, Eisenberg P, Meltzer PS, Merino MJ, Bottaro DP, Linehan WM, Srinivasan R (2013). Phase II and biomarker study of the dual MET/VEGFR2 inhibitor foretinib in patients with papillary renal cell carcinoma. J Clin Oncol.

[12] Claes A, Schuuring J, Boots-Sprenger S, Hendriks-Cornelissen S, Dekkers M, van der Kogel AJ, Leenders WP, Wesseling P, Jeuken JW (2008). Phenotypic and genotypic characterization of orthotopic human glioma models and its relevance for the study of anti-glioma therapy. Brain Pathol.

[13] Corso S, Giordano S (2013). Cell-autonomous and non-cell-autonomous mechanisms of HGF/MET-driven resistance to targeted therapies: from basic research to a clinical perspective. Cancer Discov.

[14] Ferracini R, Longati P, Naldini L, Vigna E, Comoglio PM (1991). Identification of the major autophosphorylation site of the Met/hepatocyte growth factor receptor tyrosine kinase. J Biol Chem.

[15] Gan HK, Cvrljevic AN, Johns TG (2013). The epidermal growth factor receptor variant III (EGFRvIII): where wild things are altered. FEBS J.

[16] Garnett J, Chumbalkar V, Vaillant B, Gururaj AE, Hill KS, Latha K, Yao J, Priebe W, Colman H, Elferink LA, Bogler O (2013). Regulation of HGF expression by DeltaEGFR-mediated c-Met activation in glioblastoma cells. Neoplasia.

[17] Gherardi E, Birchmeier W, Birchmeier C, Vande Woude G (2012). Targeting MET in cancer: rationale and progress. Nat Rev Cancer.

[18] Gherardi E, Youles ME, Miguel RN, Blundell TL, Iamele L, Gough J, Bandyopadhyay A, Hartmann G, Butler PJ (2003). Functional map and domain structure of MET, the product of the c-met protooncogene and receptor for hepatocyte growth factor/scatter factor. Proc Natl Acad Sci.

[19] Giordano S, Corso S, Conrotto P, Artigiani S, Gilestro G, Barberis D, Tamagnone L, Comoglio PM (2002). The semaphorin 4D receptor controls invasive growth by coupling with Met. Nat Cell Biol.

[20] Giordano S, Di Renzo MF, Ferracini R, Chiado-Piat L, Comoglio PM (1988). p145, a protein with associated tyrosine kinase activity in a human gastric carcinoma cell line. Mol Cell Biol.

[21] Han CB, Ma JT, Li F, Zhao JZ, Jing W, Zhou Y, Zou HW (2012). EGFR and KRAS mutations and altered c-Met gene copy numbers in primary non-small cell lung cancer and associated stage N2 lymph node-metastasis. Cancer Lett.

[22] Heukers R, Altintas I, Raghoenath S, De Zan E, Pepermans R, Roovers RC, Haselberg R, Hennink WE, Schiffelers RM, Kok RJ, van Bergen en Henegouwen PM (2014). Targeting hepatocyte growth factor receptor (Met) positive tumor cells using internalizing nanobody-decorated albumin nanoparticles. Biomaterials.

[23] Hirsch FR, Bunn PA, Herbst RS (2014). “Companion diagnostics”: has their time come and gone?. Clin Cancer Res.

[24] Humphrey PA, Zhu X, Zarnegar R, Swanson PE, Ratliff TL, Vollmer RT, Day ML (1995). Hepatocyte growth factor and its receptor (c-MET) in prostatic carcinoma. Am J Pathol.

[25] Jeffers M, Taylor GA, Weidner KM, Omura S, Vande Woude GF (1997). Degradation of the Met tyrosine kinase receptor by the ubiquitin-proteasome pathway. Mol Cell Biol.

[26] Jo M, Stolz DB, Esplen JE, Dorko K, Michalopoulos GK, Strom SC (2000). Cross-talk between epidermal growth factor receptor and c-Met signal pathways in transformed cells. J Biol Chem.

[27] Joffre C, Barrow R, Menard L, Calleja V, Hart IR, Kermorgant S (2011). A direct role for Met endocytosis in tumorigenesis. Nat Cell Biol.

[28] Knudsen BS, Gmyrek GA, Inra J, Scherr DS, Vaughan ED, Nanus DM, Kattan MW, Gerald WL, Vande Woude GF (2002). High expression of the Met receptor in prostate cancer metastasis to bone. Urology.

[29] Komada M, Hatsuzawa K, Shibamoto S, Ito F, Nakayama K, Kitamura N (1993). Proteolytic processing of the hepatocyte growth factor/scatter factor receptor by furin. FEBS Lett.

[30] Kong-Beltran M, Seshagiri S, Zha J, Zhu W, Bhawe K, Mendoza N, Holcomb T, Pujara K, Stinson J, Fu L, Severin C, Rangell L, Schwall R, Amler L, Wickramasinghe D, Yauch R (2006). Somatic mutations lead to an oncogenic deletion of met in lung cancer. Cancer Res.

[31] Krieger E, Koraimann G, Vriend G (2002). Increasing the precision of comparative models with YASARA NOVA–a self-parameterizing force field. Proteins.

[32] Lee CC, Yamada KM (1994). Identification of a novel type of alternative splicing of a tyrosine kinase receptor. Juxtamembrane deletion of the c-met protein kinase C serine phosphorylation regulatory site. J Biol Chem.

[33] Li L, Puliyappadamba VT, Chakraborty S, Rehman A, Vemireddy V, Saha D, Souza RF, Hatanpaa KJ, Koduru P, Burma S, Boothman DA, Habib AA (2015). EGFR wild type antagonizes EGFRvIII-mediated activation of Met in glioblastoma. Oncogene.

[34] Longati P, Bardelli A, Ponzetto C, Naldini L, Comoglio PM (1994). Tyrosines1234–1235 are critical for activation of the tyrosine kinase encoded by the MET proto-oncogene (HGF receptor). Oncogene.

[35] Lu KV, Chang JP, Parachoniak CA, Pandika MM, Aghi MK, Meyronet D, Isachenko N, Fouse SD, Phillips JJ, Cheresh DA, Park M, Bergers G (2012). VEGF inhibits tumor cell invasion and mesenchymal transition through a MET/VEGFR2 complex. Cancer Cell.

[36] Ma PC, Jagadeeswaran R, Jagadeesh S, Tretiakova MS, Nallasura V, Fox EA, Hansen M, Schaefer E, Naoki K, Lader A, Richards W, Sugarbaker D, Husain AN, Christensen JG, Salgia R (2005). Functional expression and mutations of c-Met and its therapeutic inhibition with SU11274 and small interfering RNA in non-small cell lung cancer. Cancer Res.

[37] Ma PC, Kijima T, Maulik G, Fox EA, Sattler M, Griffin JD, Johnson BE, Salgia R (2003). c-MET mutational analysis in small cell lung cancer: novel juxtamembrane domain mutations regulating cytoskeletal functions. Cancer Res.

[38] Ma PC, Tretiakova MS, MacKinnon AC, Ramnath N, Johnson C, Dietrich S, Seiwert T, Christensen JG, Jagadeeswaran R, Krausz T, Vokes EE, Husain AN, Salgia R (2008). Expression and mutational analysis of MET in human solid cancers. Genes Chromosom Cancer.

[39] Ma Q, Zhang K, Guin S, Zhou YQ, Wang MH (2010). Deletion or insertion in the first immunoglobulin-plexin-transcription (IPT) domain differentially regulates expression and tumorigenic activities of RON receptor tyrosine kinase. Mol Cancer.

[40] Mark MR, Lokker NA, Zioncheck TF, Luis EA, Godowski PJ (1992). Expression and characterization of hepatocyte growth factor receptor-IgG fusion proteins. Effects of mutations in the potential proteolytic cleavage site on processing and ligand binding. J Biol Chem.

[41] Mu FT, Callaghan JM, Steele-Mortimer O, Stenmark H, Parton RG, Campbell PL, McCluskey J, Yeo JP, Tock EP, Toh BH (1995). EEA1, an early endosome-associated protein. EEA1 is a conserved alpha-helical peripheral membrane protein flanked by cysteine “fingers” and contains a calmodulin-binding IQ motif. J Biol Chem.

[42] Navis AC, Bourgonje A, Wesseling P, Wright A, Hendriks W, Verrijp K, van der Laak JA, Heerschap A, Leenders WP (2013). Effects of dual targeting of tumor cells and stroma in human glioblastoma xenografts with a tyrosine kinase inhibitor against c-MET and VEGFR2. PLoS One.

[43] Onozato R, Kosaka T, Kuwano H, Sekido Y, Yatabe Y, Mitsudomi T (2009). Activation of MET by gene amplification or by splice mutations deleting the juxtamembrane domain in primary resected lung cancers. J Thorac Oncol.

[44] Orian-Rousseau V, Chen L, Sleeman JP, Herrlich P, Ponta H (2002). CD44 is required for two consecutive steps in HGF/c-Met signaling. Genes Dev.

[45] Ou SH, Kwak EL, Siwak-Tapp C, Dy J, Bergethon K, Clark JW, Camidge DR, Solomon BJ, Maki RG, Bang YJ, Kim DW, Christensen J, Tan W, Wilner KD, Salgia R, Iafrate AJ (2011). Activity of crizotinib (PF02341066), a dual mesenchymal-epithelial transition (MET) and anaplastic lymphoma kinase (ALK) inhibitor, in a non-small cell lung cancer patient with de novo MET amplification. J Thorac Oncol.

[46] Ozawa T, Brennan CW, Wang L, Squatrito M, Sasayama T, Nakada M, Huse JT, Pedraza A, Utsuki S, Yasui Y, Tandon A, Fomchenko EI, Oka H, Levine RL, Fujii K, Ladanyi M, Holland EC (2010). PDGFRA gene rearrangements are frequent genetic events in PDGFRA-amplified glioblastomas. Genes Dev.

[47] Paugh BS, Zhu X, Qu C, Endersby R, Diaz AK, Zhang J, Bax DA, Carvalho D, Reis RM, Onar-Thomas A, Broniscer A, Wetmore C, Zhang J, Jones C, Ellison DW, Baker SJ (2013). Novel oncogenic PDGFRA mutations in pediatric high-grade gliomas. Cancer Res.

[48] Peschard P, Fournier TM, Lamorte L, Naujokas MA, Band H, Langdon WY, Park M (2001). Mutation of the c-Cbl TKB domain binding site on the Met receptor tyrosine kinase converts it into a transforming protein. Mol Cell.

[49] Petrelli A, Gilestro GF, Lanzardo S, Comoglio PM, Migone N, Giordano S (2002). The endophilin-CIN85-Cbl complex mediates ligand-dependent downregulation of c-Met. Nature.

[50] Ponzetto C, Bardelli A, Zhen Z, Maina F, dalla Zonca P, Giordano S, Graziani A, Panayotou G, Comoglio PM (1994). A multifunctional docking site mediates signaling and transformation by the hepatocyte growth factor/scatter factor receptor family. Cell.

[51] Saikali S, Avril T, Collet B, Hamlat A, Bansard JY, Drenou B, Guegan Y, Quillien V (2007). Expression of nine tumour antigens in a series of human glioblastoma multiforme: interest of EGFRvIII, IL-13Ralpha2, gp100 and TRP-2 for immunotherapy. J Neurooncol.

[52] Schafer W, Stroh A, Berghofer S, Seiler J, Vey M, Kruse ML, Kern HF, Klenk HD, Garten W (1995). Two independent targeting signals in the cytoplasmic domain determine trans-Golgi network localization and endosomal trafficking of the proprotein convertase furin. EMBO J.

[53] Smyth EC, Sclafani F, Cunningham D (2014). Emerging molecular targets in oncology: clinical potential of MET/hepatocyte growth-factor inhibitors. Onco Targets Ther.

[54] Sottoriva A, Spiteri I, Piccirillo SG, Touloumis A, Collins VP, Marioni JC, Curtis C, Watts C, Tavare S (2013). Intratumor heterogeneity in human glioblastoma reflects cancer evolutionary dynamics. Proc Natl Acad Sci.

[55] Spigel DR, Ervin TJ, Ramlau RA, Daniel DB, Goldschmidt JH, Blumenschein GR, Krzakowski MJ, Robinet G, Godbert B, Barlesi F, Govindan R, Patel T, Orlov SV, Wertheim MS, Yu W, Zha J, Yauch RL, Patel PH, Phan SC, Peterson AC (2013). Randomized phase II trial of Onartuzumab in combination with erlotinib in patients with advanced non-small-cell lung cancer. J Clin Oncol.

[56] Tempest PR, Stratton MR, Cooper CS (1988). Structure of the met protein and variation of met protein kinase activity among human tumour cell lines. Br J Cancer.

[57] Tian S, Simon I, Moreno V, Roepman P, Tabernero J, Snel M, van’t Veer L, Salazar R, Bernards R, Capella G (2013). A combined oncogenic pathway signature of BRAF, KRAS and PI3KCA mutation improves colorectal cancer classification and cetuximab treatment prediction. Gut.

[58] Verhaak RG, Hoadley KA, Purdom E, Wang V, Qi Y, Wilkerson MD, Miller CR, Ding L, Golub T, Mesirov JP, Alexe G, Lawrence M, O’Kelly M, Tamayo P, Weir BA, Gabriel S, Winckler W, Gupta S, Jakkula L, Feiler HS, Hodgson JG, James CD, Sarkaria JN, Brennan C, Kahn A, Spellman PT, Wilson RK, Speed TP, Gray JW, Meyerson M, Getz G, Perou CM, Hayes DN, Cancer Genome Atlas Research N (2010). Integrated genomic analysis identifies clinically relevant subtypes of glioblastoma characterized by abnormalities in PDGFRA, IDH1, EGFR, and NF1. Cancer Cell.

[59] Viana-Pereira M, Lopes JM, Little S, Milanezi F, Basto D, Pardal F, Jones C, Reis RM (2008). Analysis of EGFR overexpression, EGFR gene amplification and the EGFRvIII mutation in Portuguese high-grade gliomas. Anticancer Res.

[60] Vriend G (1990). WHAT IF: a molecular modeling and drug design program. J Mol Graph.

[61] Xia N, An J, Jiang QQ, Li M, Tan J, Hu CP (2013). Analysis of EGFR, EML4-ALK, KRAS, and c-MET mutations in Chinese lung adenocarcinoma patients. Exp Lung Res.

[62] Zhang YW, Su Y, Lanning N, Gustafson M, Shinomiya N, Zhao P, Cao B, Tsarfaty G, Wang LM, Hay R, Vande Woude GF (2005). Enhanced growth of human met-expressing xenografts in a new strain of immunocompromised mice transgenic for human hepatocyte growth factor/scatter factor. Oncogene.

